# Vascular Calcification in Chronic Kidney Disease: An Update and Perspective

**DOI:** 10.14336/AD.2021.1024

**Published:** 2022-06-01

**Authors:** Si-Chong Ren, Nan Mao, Si Yi, Xin Ma, Jia-Qiong Zou, Xiaoqiang Tang, Jun-Ming Fan

**Affiliations:** ^1^Chengdu Medical College, Chengdu, China.; ^2^Department of Nephrology, First Affiliated Hospital of Chengdu Medical College, Chengdu, China.; ^3^Center for Translational Medicine, Sichuan Academy of Traditional Chinese Medicine, Chengdu, China.; ^4^Clinical Research Center for Geriatrics of Sichuan Province, Chengdu, China.; ^5^Key Laboratory of Birth Defects and Related Diseases of Women and Children of Ministry of Education, State Key Laboratory of Biotherapy, West China Second University Hospital, Sichuan University, Chengdu, China

**Keywords:** vascular calcification, chronic kidney disease, aging, metabolism, biomarker

## Abstract

Chronic kidney disease is a devastating condition resulting from irreversible loss of nephron numbers and function and leading to end-stage renal disease and mineral disorders. Vascular calcification, an ectopic deposition of calcium-phosphate salts in blood vessel walls and heart valves, is an independent risk factor of cardiovascular morbidity and mortality in chronic kidney disease. Moreover, aging and related metabolic disorders are essential risk factors for chronic kidney disease and vascular calcification. Marked progress has been recently made in understanding and treating vascular calcification in chronic kidney disease. However, there is a paucity of systematic reviews summarizing this progress, and investigating unresolved issues is warranted. In this systematic review, we aimed to overview the underlying mechanisms of vascular calcification in chronic kidney diseases and discuss the impact of chronic kidney disease on the pathophysiology of vascular calcification. Additionally, we summarized potential clinical diagnostic biomarkers and therapeutic applications for vascular calcification with chronic kidney disease. This review may offer new insights into the pathogenesis, diagnosis, and therapeutic intervention of vascular calcification.

## 1. Introduction

Vascular calcification (VC), an ectopic deposition of calcium phosphate salts in the walls of blood vessels and heart valves, is a detrimental indicator of aging-related pathological progression including diabetes mellitus, obesity, hypertension, metabolic syndrome, and chronic kidney disease (CKD) [1-3]. Clinical investigations have revealed that VC has long-term negative effects on humans and damages different arteries, including the coronary, abdominal, iliac, and femoral arteries [4, 5]. Studies on VC mechanisms have demonstrated that VC comprises a passive process of cell death combined with hydroxyapatite mineral infiltration in vessels. However, numerous recent studies have revealed that VC is an active process termed “osteogenesis,” activating vascular cells to trans-differentiate into osteoblast-like cells and inducing extracellular matrix biomineralization [6, 7]. A pathological study on VC has indicated that it can be categorized into two major types: intima and medial calcification [8]. The former is related to atherosclerosis caused by lipid and cholesterol accumulation under the injured endothelium [9], whereas the latter is induced from mineral depositions within vascular smooth muscle cells (VSMCs) and is exacerbated by aging and CKD progression [10]. Here, we focused on medial calcification induced by aging-related CKD.

CKD is a progressive irreversible renal function loss persisting for >3 months and is classified into five stages (CKD1-CKD5) according to the degree of residual kidney function and estimated glomerular filtration rate (eGFR). The most severe form of CKD is an end-stage renal disease (ESRD), caused by metabolism disorders of the proteinuria, amino acids, calcium, phosphate, and hormone homeostasis, which trigger VC processes [11]. CKD incidence increases with age, and approximately 60% of people aged ≥80 years experience it [12]. Accordingly, senescent cells in the kidney tissue increase with both age and CKD, inducing gradual kidney function loss, identified by albuminuria levels and eGFR [13]. Decreased renal function evokes metabolism disorders, including uremia, hyperphosphatemia, tubular interstitial sodium accumulation, and vitamin D and klotho protein deficiency, reciprocally promoting cell damage and senescence in kidney tissue and consequently forming vicious feedback power to exacerbate VC with CKD progression [14, 15]. These dysregulated metabolites circulate in the cardiovascular system and activate VSMC osteogenesis signaling, provoking contractible VSMCs to transdifferentiate into osteoblast-like and/or chondroblast -like cells and inducing medial calcification [2]. Recent evidence has suggested that CKD is an aging-related disease that synergizes “pro-aging” molecules aggravating VC [3]. Cellular senescence and senescence-associated secretory phenotypes (SASPs) have been reported to induce VC through VSMC pathophysiological osteogenesis mediation [16, 17]. Additionally, a study has shown that prelamin A may enhance SASP and accelerate VC processes [18]. SASP and prelamin A may also worsen CKD progression. However, the coordination between pro-aging mechanisms and CKD to accelerate VC has not been fully elucidated. We provided an overview of intricate VC mechanisms and illustrated how CKD combined with aging effects affects the pathophysiological processes of VC. Clinically measuring VC processes before diagnosis using modern imaging techniques remains arduous, which impedes disease prevention [19]. Moreover, there is a paucity of effective therapeutic applications for VC combined with CKD. Accordingly, sensitive potential VC biomarkers and more effective therapeutic regimens are required. Therefore, we also illustrated potential clinical diagnostic biomarkers and therapeutic approaches for VC combined with CKD.

**Figure 1.**
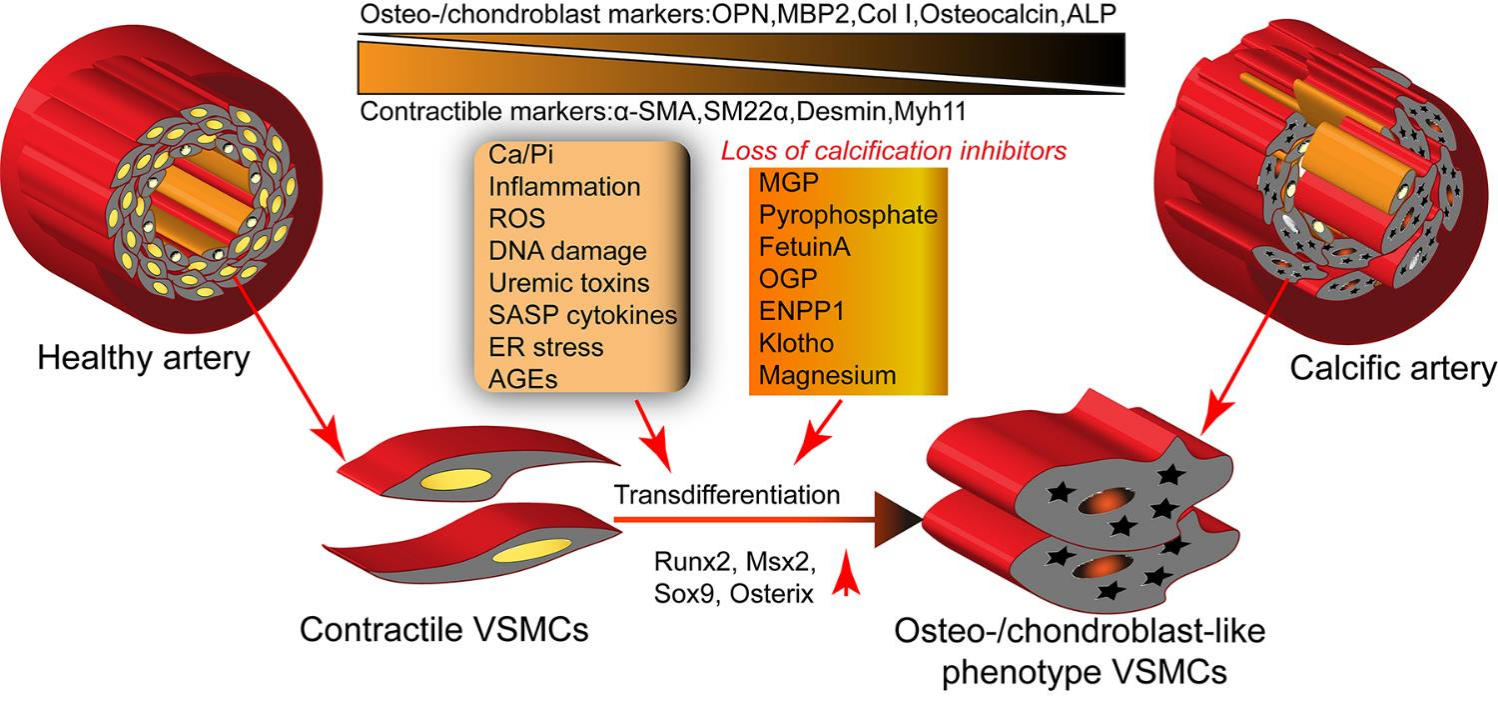
VSMC osteo/chondrogenic transdifferentiation is the key mechanism of vascular calcification. Under adverse stimuli, including Ca/Pi, inflammation, ROS, DNA damage, uremic toxins, SASP cytokines, ER stress, and age, VSMCs in the arterial wall trans-differentiate into osteo-/chondroblast cells combined with increased levels of the osteogenic master transcription factors such as Runx2, Msx2, Sox9, and osterix. This process is aggravated by the loss of calcification inhibitors, including MGP, pyrophosphate, fetuin-A, OGP, and ENPP1. These trans-differentiated VSMCs acquire osteo/chondroblast markers, type I collagen, osteocalcin, and ALP but lose contractile markers, including α-SMA and SM22-α.

**Figure 2.**
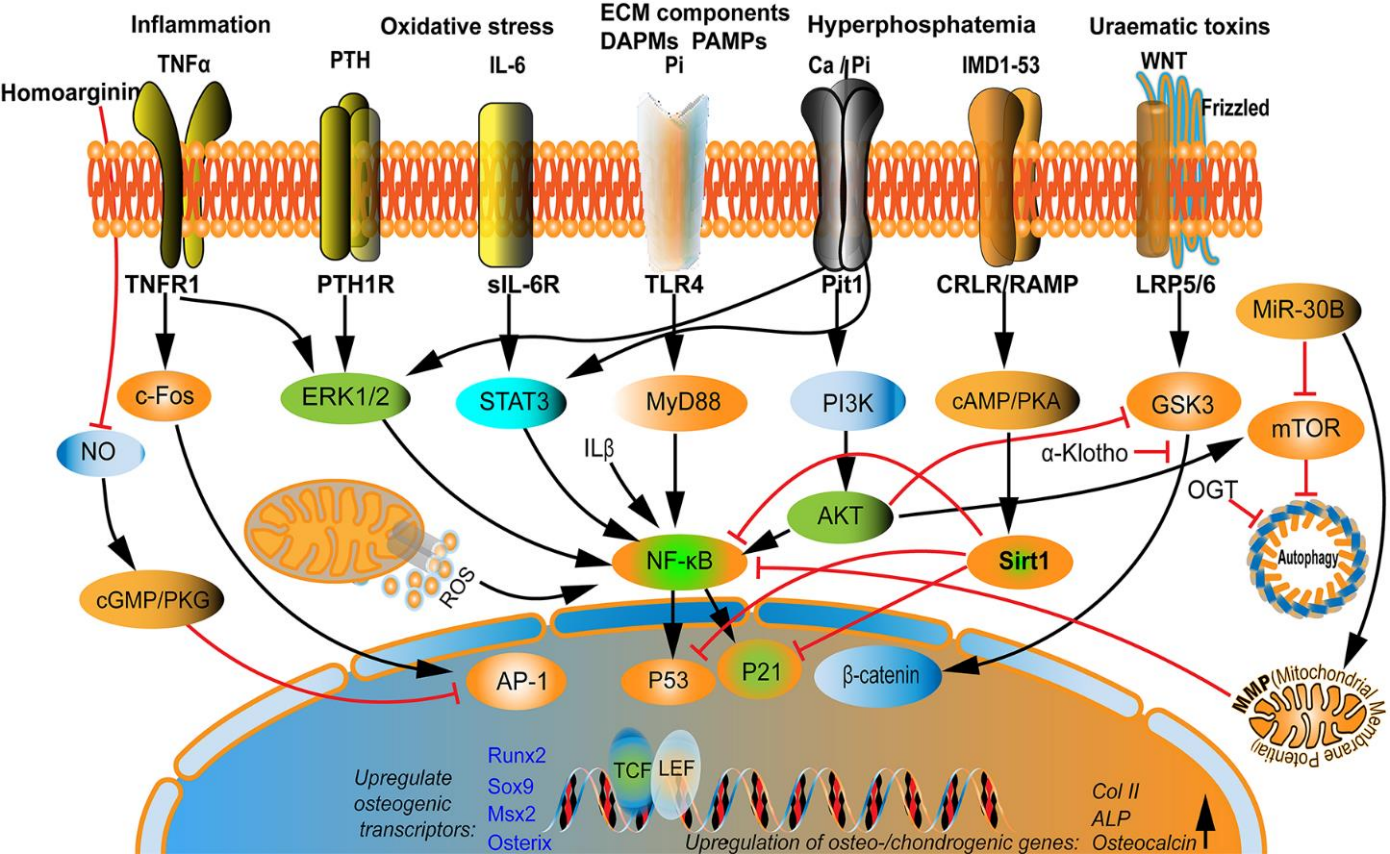
Multifactorial signaling is involved in VSMC osteo/chondrogenic transdifferentiation regulation. Various molecules directly or indirectly mediate the VSMC osteo/chondrogenic transdifferentiation process via cross-talking interaction. For example, phosphate and ECM components activate the TLR4/NF-κB signaling pathway to upregulate the osteogenic transcriptional factors Runx2 and BMP2. Moreover, calcium and phosphate activate the Pit-1 signaling pathway and interact with the IL-6 and PTH signaling pathways. PTH binds to PTH1R and ignites NF-κB signaling pathways through ERK1/2, integrating TNF-α stimulation. Additionally, TNF-α stimulates AP-1 via c-FOS to increase osteo-/chondrogenic genes expression, coordinating the IL-6/sIL-6R/STAT3/p53/p21 pathways to trigger VSMC transdifferentiation. As an interfering agent, IMD1-53 upregulates Sirt1 by activating the cAMP/PKA signaling pathway or upregulating α-klotho levels via the CRLR/RAMP3 complex to inhibit Wnt/β-catenin signaling. miR-30B promotes the MMP and autophagy-related to the mTOR signal pathway, and crosstalk exists between this signaling pathway and NF-κB. PTH, parathyroid hormone; TLR4, toll-like receptor 4; ERK1/2, Extracellular regulated protein kinases 1/2; PTH1R, parathyroid hormone 1 receptor; sIL-6R, soluble IL-6 receptor; STAT3, signal transducer and activator of transcription 3; IMD1-53, intermedin 1-53; CRLR, calcitonin receptor-like receptor; RAMP3, receptor activity-modifying protein-3; and MMP, mitochondrial membrane potential.

## 2. Overview of intricate VC mechanisms

VC is an actively regulated biological process involving ectopic depositions of crystalline hydroxyapatite in arterial walls and shares numerous features with embryonic bone ossification. VSMC is the dominant mediator in the aforementioned deposition process that enables osteon/chondrogenic transdifferentiation programs to respond to multifaceted adverse pathogenic factors [20]. Hyperphosphatemia, hypercalcemia, uremic toxins, and other harmful biological factors, including oxidative stress, DNA damage, and inflammatory cytokines, are intensive stimuli for VSMC osteogenesis and progression [21, 22]. This study summarizes the effects of VSMCs and multifarious factors on VC pathogenesis and progression, respectively.

### 2.1. Maladaptive VSMCs involved in VC pathogenesis

The following three events are key factors confirming VC progression: osteon/chondrogenic VSMC trans-differentiation; formation of a nucleated calcium-phosphate mineral structure; and calcium-phosphate nanocrystal endocytosis. These complex maladaptive modulation processes tend to occur simultaneously and not in a stepwise manner [2]. Osteo/chondrogenic transdifferentiation is initiated by the upregulation of osteogenic master transcriptional factors, including RUNX2, SOX9, MSX2, and osterix, when VSMCs inadequately respond to pro-calcifying stimulations, thus inducing the expression of downstream osteogenic mediators, including type I collagen, alkaline phosphatase, osteopontin, and osteocalcin (Fig. 1) [2, 10]. Nevertheless, this process is regulated by various signal pathways. Studies have delineated that the NF-κB, TNF-α, Wnt/β-catenin, and PI3K signaling pathways are engaged in VSMC osteogenesis under various pro-calcifying stimulations [23-26]. GSK-3, a serine/ threonine kinase, is a component of the APC/AXIN/GSK3 complex in the cytoplasm involved in the regulation of the Wnt/β-catenin signaling pathway [27, 28]. Activated PI3K/AKT signaling pathways inhibit GSK-3β activity and release β-catenin from the APC/AXIN/GSK3 complex, enhancing β-catenin nucleus translocation and activation. Furthermore, activated β-catenin cells coordinate with T-cell or lymphocyte enhancer factors to upregulate osteogenic gene expression [29]. Pit-1 is a cellular membrane channel protein and a sodium-dependent phosphate co-transporter, mediating phosphate and calcium translocation from the extracellular environment into the intracellular compartment. Hyperphosphatemia or hypercalcemia may induce Pit-1 expression in VSMCs and activate Pit-1 signaling to mediate osteogenic programs [30, 31]. Moreover, the toll-like receptor 4 (TLR4) signaling pathway may respond to phosphate stimulation and initiate the phenotypic switch of VSMC osteoblast-like cells [32]. Parathyroid hormones (PTHs) are secreted by the chief cells of the parathyroid, regulating calcium hemostasis. Studies have suggested that PTHs directly target PTH 1 receptors and negatively affect VSMC osteogenesis [33]. Other signaling pathways, including sIL6 and miRNA, and small metabolites also contribute to transdifferentiation. These pathways respond to various stimuli and induce molecular crosstalk between them in a fine modulated signaling network, mediating VSMC osteo/chondrogenic transdifferentiation (Fig. 2).

These trans-differentiated VSMCs decrease the expression of the α-SMA and SM22-α contractile markers while acquiring synthetic features. Moreover, they synthesize and release small calcifying vesicles of 30-300 nm in diameter, forming calcium-phosphate crystal nucleus and inducing extracellular matrix mineralization. Secreted matrix vesicles act as original sites for hydroxyapatite crystal nucleated structures and their pro-calcifying contents, namely, dying apoptotic bodies, necrosis debris, endosomes, autophagosomes, and enriched ALP [34-36]. Nevertheless, these matrix vesicles induce the loss of mineralization inhibitors, including fetuin-A and matrix Gla protein (MGP). Furthermore, the degradation of extracellular matrix fibronectin, elastin, and collagens is induced by matrix metalloproteinase and collagenases [37]. These pro-calcifying vesicles and degraded extracellular matrix fragments precipitate calcium-phosphate salts and synergistically form nucleated crystal structures [38]. The endocytosis of calcification matrix vesicles by neighboring VSMCs extend hydroxyapatite deposition and facilitate nanocrystal formation, worsening calcification in the arterial walls. *In-vivo* and *in-vitro* experimental data have shown that these processes may occur simultaneously and be modulated by various signaling pathways such as mTOR, Annexin, and miRNAs, activated by extracellular matrix, inorganic milieu, and vesicle contents [30, 39-41].

### 2.2. Multifarious factors affecting VC progression

VC is an active process modulated by multifarious factors. Epidemiological studies have shown that serum phosphate, calcium, PTH, and vitamin D mediate the expression of VSMC osteo-/chondrogenic genes, matrix vesicle release, and hydroxyapatite deposition [32, 42, 43]. In contrast, numerous endogenous inhibitors, including MGP, fetuin-A, klotho, and pyrophosphate (PPi), significantly contribute to anti-ectopic calcium-phosphate salt deposition [44, 45]. MGP is a vitamin K-dependent matrix Gla protein expressed in the bones, heart, vessels, and kidneys [46, 47]. It serves as a strong VC inhibitor by directly binding with calcium-phosphate particles to impede hydroxyapatite mineral formation and hampers VSMC osteoblastic switching by deactivating the BMP-2 signaling pathway [48]. Moreover, MGP removes extracellular matrix microcalcification vesicles before their deposition in vessel walls [49]. This finding was supported by a study demonstrating that MGP knock-out mice exhibited extensive spontaneous artery calcification and died within 2 months [50, 51]. Fetuin-A is a negative charge glycoprotein mostly released by the liver, identified as a VC inhibitor that forms large calciprotein particles (CPPs) and prevents continual mineral crystal deposition by directly binding to calcium apatite [52, 53]. Additionally, fetuin-A protects VSMCs from osteoblastic phenotype switches and suppresses apoptotic body and inflammatory cytokine release[54, 55]. Recent studies have demonstrated that “klotho,” an anti-aging protein, inhibits VC processes primarily through phosphate reabsorption repression using the renal tubule, thus inducing phosphatidic and decreasing calcitriol synthesis [56, 57]. Mice with klotho deficiency have a short life span, premature aging, and ectopic calcification [58], whereas those supplied with klotho show reduced serum phosphate levels and VC progression and partially remedied adverse phenotypes [59]. Moreover, klotho may be a coreceptor concerted with FGF-23, another calcification inhibitor secreted by osteocytes in bones, to modulate calcification. FGF23 acts as a circulating ligand directly bound to klotho on renal tubular epithelial cells, thus enhancing phosphate excretion by reducing phosphate reabsorption and sodium/phosphate transporter NPT2a and NPT2c expression [60, 61]. Unlike the abovementioned proteins, PPi, a key inorganic endogenous VC inhibitor, is produced from extracellular ATP. Studies have revealed that extracellular ATP hydrolysis by ectonucleotide pyrophosphatase (ENPP) is the main resource of PPi, and any additional supplements from intracellular substances are transported out of the cell by the membrane transporter ankylosis protein homolog (ANKH)[62, 63]. Amorphous hydroxyapatite formation is strongly inhibited by extracellular PPi [62]. Recent data have shown that sufficient physiological PPi concentration may inhibit calcification when serum phosphate levels are at a normal range, but may fail to do so if hyperphosphatemia is present [64]. ENPP1 hydrolyzes extracellular ATP to generate AMP and PPi; therefore, AMP can be degraded to adenosine and PPi to ecto-5′nucletotidase [65]. Moreover, tissue-nonspecific alkaline phosphatase degrades PPi into phosphate ions. Accordingly, the Pi to PPi ratio, engaged enzyme levels, and activity states are considered equally contributing factors for VC processes [66, 67].

Contrary to endogenous inhibitors, various biologic events, including inflammation, oxidative stress, and ER stress, promote VC processes. Inflammatory cytokines accelerate VC. Studies have indicated that serum, glucocorticoid-inducible kinase 1, C-reactive protein (CRP), and Fc region receptor II-a may activate the NF-κB signaling pathway to augment the osteo-/chondrogenic transdifferentiation of VSMCs [2, 68-70]. Similarly, oxidative stress triggers the phenotypic switch of VSMCs by prominently upregulating Runx2 and ALP expression [71]. In contrast, the suppression of ROS products, inhibition of mitochondrial reactive oxygen species, and augmentation of anti-oxidative enzyme SODs may partly reverse VC processes [72, 73]. Studies have revealed that kidney metabolism disorders in patients with CKD enhance inflammatory and ROS signaling, while impaired VC inhibitory signaling, in coordination with the aforementioned disorders, prominently exacerbates VC progression [44, 74, 75]. Additionally, recent studies have found that the ER stress-activated CHOP-ATF4 signaling pathway contributes to the transdifferentiation of VSMCs and VC progression [76]. Furthermore, it has been suggested that amino acid metabolites, including homoarginine, indoxyl sulfate (IS), and trimethylamine-N-Oxide, affect VC through various signaling pathways [77-79]. VC is a complex and multifactorial biologic process, and numerous molecules are involved in mediating it through various signaling pathways and targets, leading to its different effects. This study briefly summarizes the molecules involved in VC pathogenesis and elucidates their engaged signaling in regulating VC progression (Table 1).

**Table 1:** Molecules involved in VC process.

Classification	Molecules	Targets	Effects	Involved tissues/cells	Comments	Ref.
Mineral metabolism	Calcium	Pit-1	VC exacerbation	Kidneys, vessels, bones, and intestinal tract	Induces the expression of Pit-1	[43]
	Phosphate	TLR4	VC exacerbation	Kidneys, vessels, bones, and intestinal tract	Activates the TLR4/NF-κB signaling pathway	[32, 113]
	IGF2	Klotho	VC suppression	Kidneys, vessels, and bones	Reduces inflammation and oxidative stress and affects klotho expression	[234, 235]
Hormones	PTH	PTH1R	VC exacerbation	Kidney, vessels, bone, and parathyroid glands	Activates the ERK1/2 and NF-κB signaling pathways	[42]
	AT2	RAS	VC alleviation	Kidneys, vessels, and bones	Stimulates PPAR-γ through klotho expression upregulation	[117, 236]
	Estrogen	HIF-1	VC alleviation	Vessels and bones	Affects BMP-2-p-Smad1/5/8 signaling	[237]
	Growth hormone-releasing hormone	NF-κB, PKA	VC alleviation	Hypothalamus, pituitary, vessels, and bones	Cross talking between the RANKL-NFκB-Runx2 and GHRHR-cAMP-PKA signaling pathways	[238]
Inflammation	IL-6	p53	VC exacerbation	VSMCs	Activates the IL-6/sIL-6R/STAT3/p53/p21 pathway	[25, 239, 240]
	IL-1β	p53	VC exacerbation	VSMCs	Activates the NF-κB/p53/p21 pathway	[144]
	TNF-α	AP-1	VC exacerbation	VSMCs	Activates the TNF- α-AP-1/c-FOS signaling axis	[26]
MicroRNAs	miR-204	DNMT3a	VC alleviation	VSMCs	Affects the MiR-204/DNMT3a regulatory circuit	[192]
	miR-30B	SOX9	VC alleviation	VSMCs	Activates the MMP and autophagy involved in the mTOR signaling pathway	[191]
	miR-135A	KLF-4, STAT3	VC alleviation	VSMCs	Affects the KLF-4/STAT3 pathway	[241]
Enzymes/Small molecules	Phospholipase D	PKC	VC exacerbation	VSMCs	Affects PKC-independent manner activation	[242]
	CDC42	AKT	VC exacerbation	VSMCs	Activates the AKT signaling pathway	[243]
	OGT	Autophagy complex	VC exacerbation	VSMCs	Inhibits autophagy through YAP upregulation	[114]
	IMD1-53	Sirt1 and klotho	VC alleviation	VSMCs	Upregulates Sirt1 by activating the PI3K/Akt and cAMP/PKA signaling pathways and increases α-klotho via the CRLR/RAMP3 complex	[153, 154]
	LGR4	NF-κB	VC exacerbation	Kidneys, vessels, bones, and parathyroid gland	Activates the PTH/PKA signaling pathway	[23]
	MGP	Vitamin K	VC alleviation	Kidneys, vessels, and bones	Activated by vitamin K dependent γ-carboxylation	[45, 163, 244]
	Lamin A	RUNX2	VC exacerbation	VSMCs	Interacts with RUNX2 and facilitates their nuclear localization	[245]
Amino-acid metabolites	Homoarginine	NO	VC exacerbation	Kidney, vessels, and bones	Impairs NO production and triggers osteo-/chondrogenic signaling	[78]
	IS	Klotho, TRPM7	VC exacerbation	VSMCs	Mediates klotho gene expression or downregulates TRPM7, and induces cross-talking between OATP2B1 and Dll4-Notch axes	[79, 122, 123, 129]
	TMAO	NF-κB	VC exacerbation	VSMCs	Activates NLRP3 inflammation and NF-κB signaling	[77]

## 3. VC is a severe detrimental cardiovascular complication of CKD

CKD, a progressive and irreversible renal function loss, has one of the highest chronic disease morbidities in the world. The 2017 Global Burden of Disease Study has shown that 1.2 million people died of CKD, a number that has recently increased rapidly [80]. People with CKD often suffer from multiple comorbidities impairing their quality of life, and most of these people prematurely die, even before dialysis, owing to cardiovascular complications [81]. VC is a key pathological process contributing to cardiovascular CKD complications. It is highly prevalent in patients with CKD, particularly those with ESRD [82]. Therefore, it is a strong independent risk factor for cardiovascular events and mortality in patients with CKD [83].

VC inhibits endothelial and VSMC cells from modulating circulating circumstances, inducing artery stenosis, arterial stiffness, and high pulse pressure, which increases the risks of ischemia, hypertension, and left ventricular hypertrophy [24, 84, 85]. Various data have shown that CKD evokes calcification in the arterial system and causes detrimental outcomes. Coronary artery calcification (CAC) is highly prevalent in patients with atherosclerosis induced by lipid and ox-LDL, causing declined vascular compliance, abnormal vasomotor responses, and impaired myocardial perfusion[86]. Common carotid artery intima-media thickness (CCA-IMT) is usually considered as a CAC predictor, and a recent study has indicated that it is associated with higher incidences of medial arterial calcification and cardiovascular events in patients with CKDt [87]. Additionally, CAC is significantly related to dialysis outcomes in patients with CKD, and patients with CKD and CAC have a 60-fold higher mortality rate than those with CKD only. Therefore, researchers have suggested that the CAC score is a valid indicator for favorable long-term risk prediction and the risk stratification of patients with CKD[4, 88, 89]. Numerous findings have recently suggested that abdominal aortic calcification (AAC) is an independent predictor of all-cause mortality in patients undergoing dialysis, particularly those undergoing peritoneal dialysis [90-92]. Moreover, the AAC score may be used to assess targeted risk-lowering strategies for kidney transplant recipients [93]. In addition to CAC and AAC, other large and medium arteries, including the aortic arch, iliac artery, pelvic artery, femoral artery, and lower leg arterial calcification may also be used to predict the mortality of patients with CKD. Nevertheless, the causal relationship between these factors and mortality in patients with CKD should be further investigated [91, 94-98].

## 4. Disorders of renal metabolism contribute to VC development in patients with progressive CKD

The kidney is a pivotal organ regulating minerals and the acid-base balance through the renal tubule and duct system. The absorption and excretion of calcium, phosphate, sodium ions, magnesium, amino acids, H^+^, HCO3^-^, and PTH are important physiological kidney functions. The renal metabolism of these minerals and metabolites is essential for hemostasis. The bone is another regulator and reservoir of minerals synergizing with the kidney to maintain calcium phosphate homeostasis. Studies have demonstrated that renal metabolism dysregulation causes pathological changes, particularly by eventually initiating VC pathogenesis [99-101].

### 4.1 Renal metabolism dysregulation in patients with progressive CKD

Dysregulated calcium-phosphorus products and hypocalcemia trigger the release of PTH from parathyroid gland chief cells, resulting in bones liberating and renal tubule absorbing calcium to restore balance[102, 103]. However, in patients with physiological metabolism conditions, the kidney synthesizes 1,25-hydroxyvitamin D3, which, combined with other substances, regulates calcium absorption and PTH expression in regular calcium homeostasis [104]. Similar to calcium, magnesium hemostasis is also primarily controlled by the kidney through renal glomerular filtration and renal tubule excretion [105]. With CKD progression, PTH, vitamin D3, and other metabolites become dysregulated, causing mineral and bone metabolism disorders termed CKD-mineral and bone disorders (CKD-MBDs). CKD-MBDs significantly accelerate VC processes[106]. The striking prevalence of cardiovascular morbidity is due to CKD-related metabolism disorders, and typical disorders seemingly include hypocalcemia, hyperphosphatemia, and secondary hyperparathyroidism (sHPT) [106-108]. Hyperphosphatemia is reportedly due to decreased renal tubular excretion, subsequently stimulating PTH secretion and accelerating bone turnover and calcium deposition in arterial walls [109]. sHPT could be significantly aggravated in patients with ESRD because of vitamin D3 synthesis and calcium resorption impairment [110, 111]. Magnesium is an important metallic ion contributing to pathophysiologic events. Circulating magnesium hemostasis is reportedly disrupted as CKD progresses, although circulating magnesium levels may slightly increase due to decreased excretion [112]. Furthermore, pathogenic VC stimuli such as uremic toxins, inflammatory mediators, and ROS, are also dysregulated in patients with CKD.

### 4.2. Mechanisms of dysregulated metabolites underlying VC initiation

**Figure 3.**
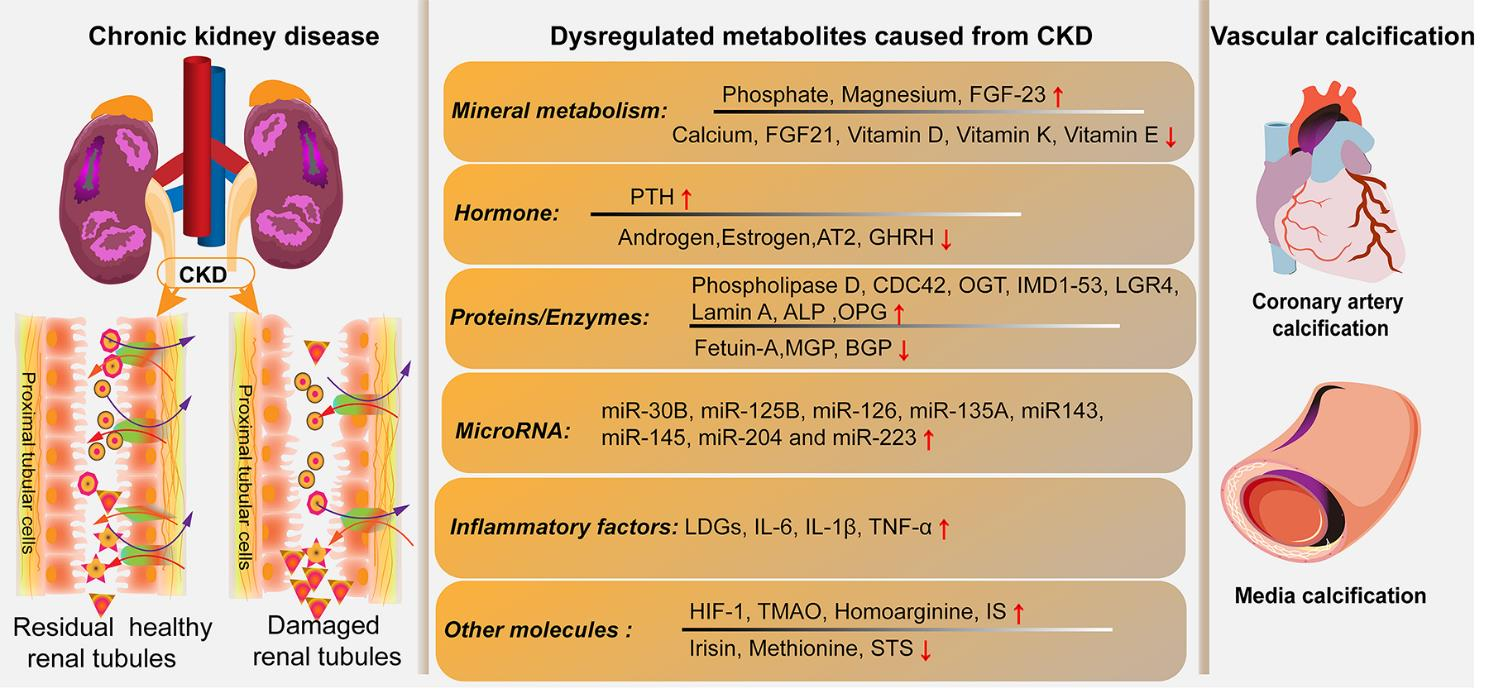
Dysregulated metabolites aggravate VC processes in patients with progressive CKD. In patients with progressive CKD, the damaged and residual healthy renal tubules lose the compensatory functions of excreting and reabsorbing metabolites. These metabolites, including mineral metabolism modulators, hormone, enzymes, microRNAs, inflammatory factors, and other molecules, lead to physiological homeostasis disorders. Consequently, these dysregulated metabolites initiate and accelerate VC processes, resulting in calcium-phosphate depositions in the media layer of central large coronary arteries and/or other peripheral aortas.

The dysregulated metabolites circulate in the vascular system and induce adverse effects on VSMCs, initiating inflammation, oxidative stress, and VC processes (Fig. 3). Studies have indicated that elevated phosphates and PTH upregulate the expression of leucine-rich repeat-containing G-protein coupled receptor 4, thus activating the PTH/PKA signal pathway [23]. Additionally, hyperphosphatemia and hypercalcemia contribute to VC processes by secreting Pit-1 proteins at the early stages of CKD, whereas the hypocalcemia induced from the aforementioned two factors initiates the shots signaling pathway in patients with ESRD [43]. Moreover, increased phosphate levels may activate the TLR4/NF-κB signaling pathways in VSMCs, generating inflammatory cytokines and exerting the phenotypic switch of osteoblast-like cells [32, 113]. O-GlcNAc transferase is a post-transcriptional modification enzyme that may be reportedly transformed into glycosylate Yes-associated proteins by hyper-phosphatemia, blocking VSMC autophagy and expediting the VC process [114]. FGF23 functions as a regulating phosphate and synthesizes vitamin D. Studies have reported that hyperphosphatemia significantly increases FGF23 expression in patients with CKD. Additionally, it is strongly associated with mortality and other VC-induced adverse outcomes; however, the exact mechanisms underlying this association remain to be elucidated [97, 115, 116]. Regarding the association between hyperphosphatemia and inflammation and oxidative stress, numerous studies have suggested the involvement of the hypoxia-inducible factor-1, nitric oxide synthesis, angiotensin II type 2 receptor, and peroxisome proliferator-activated receptor-γ signaling pathways [78, 117, 118]. Furthermore, the endogenous non-proteinogenic homoarginine metabolic signaling pathway has been reported to impair the function of nitric oxide products, causing oxidative stress and VC processes under hyperphosphatemia [78]. Magnesium metabolism usually increases in patients with CKD. Few studies have indicated that supraphysiological magnesium may inhibit VC processes through four main mechanisms as follows: inhibition of the formation of calcium phosphate dihydrate crystals, activation of VSMCs calcium-sensing receptors (CaSRs) to impair excessive Ca^2+^ uptake, enhancement and/or restoration of TRPM7 channel activity, and downregulation of the Wnt/β-catenin signaling pathway [119-121].

Furthermore, other metabolites and/or molecules have induced VC processes in patients with CKD. IS, a protein-bound uremic toxin, is increased in the circulation of patients with CKD and is positively associated with the CAC score. Studies have indicated that it inhibits the vascular klotho expression and downregulates TRPM7 levels, thus promoting VC processes [79, 122, 123]. IS, as an important pro-inflammatory molecule, may stimulate the secretion of interleukin (IL)-8 from the endothelium and impel calcium depositions by downregulating osteopontin in patients with CKD [124]. Similar results have shown that IS releases inflammatory cytokines from activated macrophages in patients with CKD, a process mediated by crosstalk signaling between the OATP2B1 and Dll4-Notch axis pathways [125]. Trimethylamine-N-Oxide, a metabolite generated by gut microbiota [126, 127], is a gut-derived uremic toxin accelerating VC processes by modulating the NLRP3 inflammatory and NF-κB signaling pathways [77].

**Figure 4.**
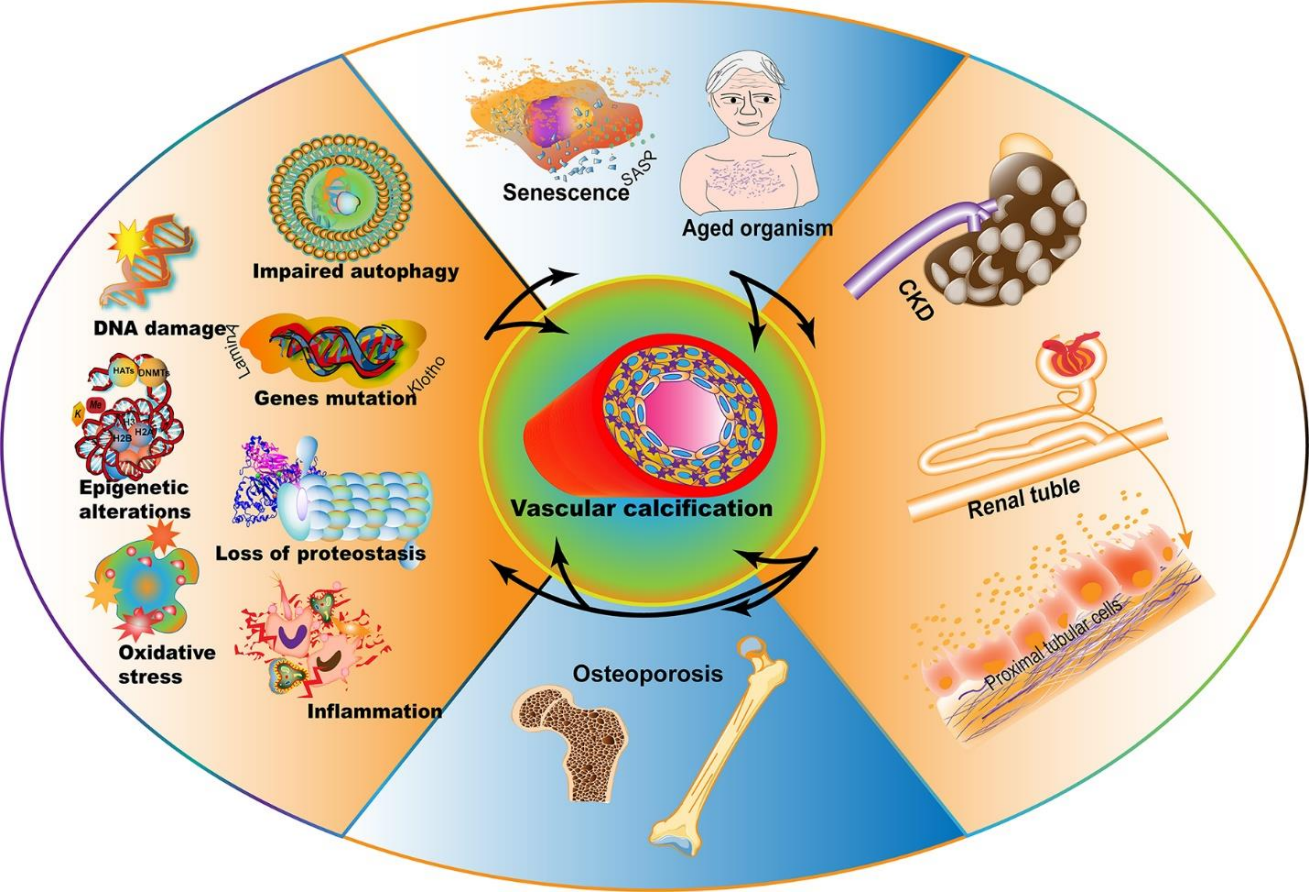
Aging as a vicious accelerator exacerbates VC progression in patients with CKD. Pro-aging factors including DNA damage, epigenetic alterations, oxidative stress, impaired autophagy, klotho and LaminA mutations, proteostasis loss, and inflammation, induce cellular senescence and aged organisms, promoting kidney aging and damage, and consequently accelerating CKD progression. The proximal tubular cells were damaged, and the excretion and absorption functions were abnormal under CKD conditions. Impaired kidney metabolism function evokes metabolite disorders, affecting bone mineralization and osteoporosis and reciprocally increasing pro-aging stimulus, which induces a vicious cycle of pro-calcifying force. Furthermore, the arc arrows indicate that these adverse pro-aging factors (aging, CKD, and osteoporosis) facilitate VC processes. Aging is a hub linker and pivotal accelerator of CKD progression, contributing to VC progression and CKD cardiovascular events. However, the mechanisms regarding this matter need further elucidation.

With CKD progression, these uremic toxins are gradually accumulated, enhancing inflammation and ROS products and forming vicious cycles that aggravate VC processes. Studies have reported dysregulated metabolites as VC accelerators, though the precise mechanisms underlying this process remain to be investigated. VSMC damage and osteogenesis phenotype switching are key pathological features of VC, initiated by the aforementioned dysregulated metabolites [44]. The reduction of calcification inhibitors, including klotho, fetuin-A, and MGP, is also related to VC processes. Additionally, risk factors exacerbating VC processes in patients with progressive CKD have been partially illustrated by clinical researchers. Epidemiological studies have indicated aging, diabetes, hypertension, cholesterol, BMI, and male gender as typical risk factors for VC in patients with CKD [128, 129]. These harmful factors synergize with dysregulated calcium, phosphate, hormone metabolites, and calcification activators to worsen complex sets of VSMC maladaptive osteo-/chondrocyte-like trans-differentiates in patients with progressive CKD (Fig. 4)[10]. Moreover, studies have indicated that maladaptive VSMC processes may be aggravated by aging. Accordingly, it has been suggested that anti-aging interventions may prevent VC in patients with CKD [130]. Therefore, elucidating how aging aggravates VC processes in patients with CKD is essential to solving problems regarding clinical interventions for VC.

## 5. Aging aggravates VC attrition in patients with progressive CKD

Aging is an irreversible process occurring in organisms affected by environmental and genetic modifications and eventually leading to numerous age-related diseases. It is characterized by progressive physiological losses, leading to impaired function and increased vulnerability to harmful detriments, including DNA damage, epigenetic alterations, genome instability, gene mutations, oxidative stress, and inflammation [131]. Recent studies have indicated that CKD is an age-related disease, and that VC increases with age. Approximately 60% of 60-year-olds and, surprisingly, up to 90% of men and 67% of women aged >70 years have VC [27]. Accordingly, CKD incidence gradually increases with aging, and the GFR for defining CKD steadily declines with normal aging, with an average reduction of approximately 0.75-1.0 ml/min/ 1.73 m2/year, beginning at approximately 30 years [132]. Nephrosclerosis, a major hallmark of all CKD types, is characterized by increased focal and/or global glomerulosclerosis, interstitial fibrosis/tubule atrophy, and arteriolosclerosis associated with aging progression [133, 134]. Investigating whether aging synergizes with CKD to aggravate VC processes is required. Insights into pathophysiological links between aging and CKD may offer investigators new perspectives for research and therapeutic applications related to VC.

Indeed, various studies have demonstrated that pro-aging factors accelerate CKD progression and VC processes, although the complicated multifactorial mechanisms underlying this association should be further elucidated [15]. Pro-aging factors, including cellular senescence, SASP, klotho gene deficiency, oxidative stress, prelaminar accumulation, and osteoporosis, reportedly aggravate VC in patients with progressive CKD [3, 135, 136].

Cellular senescence is an irreversible cell growth inhibition triggered by DNA damage, oxidative metabolites, and pro-inflammatory mediators [137]. Additionally, glomerular podocytes, terminally differentiated cells in adults, are crucial for maintaining glomerular function. The number of senescence podocytes increases with aging. Moreover, these cells are reportedly unable to adequately proliferate to replace themselves and aggravate CKD [133, 138]. Uremic toxins, including an extremely heterogeneous group of molecules, are the main source of oxidative metabolites in patients with CKD. They may forcefully initiate VC through inflammation, DNA damage, oxidative stress, and senescence [139]. CDKN2A/p16Ink4a and osteogenesis transcriptional factor Runx2 significantly increased in a rat adenine-induced uremia CKD model [140]. Furthermore, increased CDKN2A/p16INK4a and SA-β-Gal positive cells are associated with extensive VC in patients with CKD [141]. Persistent systemic inflammatory cytokines and oxidative stress products are the main initiators of premature aging and the senescence cells contributing to VC processes are commonly observed in patients with advanced CKD [142]. Similar to cell senescence, SASP is characterized by secreted factor profiles of pro-inflammatory cytokines, growth factors, and soluble oxidative stress products. SASP factors, including IL-1, IL-6, and IL-8, accelerate aging, and corresponding positive feedbacks are mediated by NF-κB, p38MAPK, and inflammation signaling pathways. Studies have shown that SASP factors, including IL-6, OPG, BMP-2, and MCP-1, regulate VSMC osteogenesis, thus accelerating VC progression in patients with CKD [15, 143]. Furthermore, IL-6 may also synergize with excess phosphates to accelerate VC depending on p53 in patients with ESRD. Similarly, IL-1β contributes to senescence-associated calcification through the NF-κB/p53/p21 signaling pathway [25, 144]. Moreover, oxidative stress products from SASP impel VC processes and CKD progression by activating the renin-angiotensin system, TGF-β, and Wnt/β-catenin signal pathways, consequently accelerating kidney fibrosis and ESRD [68, 145, 146].

Genetic adverse modifications cause aging, while deficiencies in the anti-aging gene klotho induce soft tissue calcifications, osteomalacia, and kidney malfunction [147]. Klotho is expressed primarily in renal distal convoluted tubules and in chief parathyroid cells. Klotho has two isoforms: type I membrane-bound a-klotho acts as an essential FGF23 co-receptor, inhibiting VC via the FGF23-klotho signaling pathway, whereas the other secreted klotho serves as a hormone and has various biologic functions besides FGF23 [148]. Clinical studies have shown that klotho levels in kidney tissue, serum, and urine, decrease in patients with CKD and are inversely correlated with disease severity. CKD is significantly positively correlated with eGFR; thus, soluble klotho may be an early CKD biomarker and VC progression indicator [135, 149, 150]. Studies have revealed that klotho mitigates renal fibrosis and kidney failure by inhibiting mitochondrial injury [151, 152] while it may attenuate VC and ameliorate kidney function in patients with CKD partially through the CRLR/RAMP3 complex-mediated cAMP/PKA signaling pathway. The activity of this pathway is regulated by intermedin 1-53, a calcitonin/ calcitonin gene-related peptide, via mediating klotho expression [153, 154]. Moreover, klotho overexpression may attenuate renal dysfunction and artery and kidney calcification concomitantly in mice with CKD because of improved imbalances in phosphate levels and VSMC osteoblast-like phenotype switches [155, 156]. Furthermore, loss of klotho levels due to aging progresses both CKD and VC, although the corresponding mechanisms remain to be investigated. Cellular biologic events, including oxidative stress, inflammation, and epigenetic modulation, are involved in klotho gene expression dysregulation. Additionally, klotho deficiency has been strongly associated with abnormalities in phosphate and uremic toxins, which reciprocally accelerated aging and VC progression in patients with CKD [135, 157].

Other culprit mutant genes contribute to premature aging. Additionally, LMNA mutation, i.e., the Hutchinson-Gilford Progeria Syndrome, is another critical genetic disorder, inducing premature aging. The LMNA gene-coded nuclear lamina protein lamina exerts physiologic functions, while mutant LMNA induces loss of cleavage site for endopeptidase FACE1, abundantly accumulating its precursor protein prelamin A in cellular nuclei. Patients suffering from this mutation develop various aging-related phenotypes, including hair loss, short stature, osteoporosis, and death within 20 years of age [158]. It has been demonstrated that prelamin A occurs during VSMC aging and CKD progression, accelerating DNA damage and premature senescence. Furthermore, it has been found that prelamin A is located in the calcified arteries of children with dialysis, but not in normal control subjects [18]. Accordingly, a transgenic mouse model has demonstrated that prelamin A overexpression also exacerbates the VC process via BMP-2 and Runx2 upregulation to initiate VSMC osteogenesis program [159]. Additionally, oxidative stress and uremic toxins reportedly induce prelamin A accumulation in VSMCs, accelerating DNA damage, premature senescence, and consequent VC progression [10]. These studies strongly imply that prelamin A may accelerate VC in patients with progressive CKD. However, the direct and exact mechanisms underlying this link remain unclear. Moreover, the interplay among LMNA mutants, klotho deficiency, SASP, and other pro-aging factors accelerating VC progression in patients with CKD remains to be investigated. Nevertheless, these pro-aging factors induce cellular senescence, kidney injures, and function failure, consequently accelerating CKD progression. Impaired kidney metabolism function dysregulates, affecting bone mineralization, inducing osteoporosis, and increasing pro-aging stimuli to form vicious cycles of pro-calcification in patients with CKD (Fig. 4). Therefore, metabolites involved in VC processes, such as VC indicators and/or biomarkers in patients with CKD, should be investigated. Moreover, diagnosing VC and interrupting its progression at early periods in such cases, may be beneficial.

## 6. Diagnostic biomarkers of VC in patients with CKD

The high prevalence of cardiovascular events induces huge medical costs and poses enormous threats to public health. Accordingly, valid diagnostic biomarkers for the early detection of VC are warranted. Various imaging techniques, including radiography, computed tomography, magnetic resonance imaging, and ultrasonography, have been applied to qualitatively and quantitatively assess VC; however, these approaches identify only locally a macrocalcification lesion in aortic walls. Therefore, measurements for estimating the severity of microcalcification processes are essential for clinical diagnoses and interventions. These ideal diagnostic surrogates should be noninvasive, economical, quantitative, specific, and sensitive for distinguishing ectopic mineralizations in arterial walls. Recent evidence has suggested that serum circulating molecules are involved in the regulation of mineral metabolism and ectopic calcification processes, including MGP, fetuin-A, FGF-23/klotho, PPi, and sclerostin, are favorable diagnostic biomarkers for VC in patients with CKD [19, 160]. We herein present these excellent candidate clinical biomarkers for VC diagnosis.

### 6.1. MGP

MGP is a strong VC inhibitor, whose activity depends on the carboxylation of Glu with γ-glutamyl, catalyzed by γ-glutamyl carboxylation with vitamin K as a co-factor for converting Glu to γ-carboxyglutamic acids (Gla)[54]. Another post-translational modification is serine residue phosphorylation, also comprising MGP activity. Phosphorylated MGP strongly tends to negatively neutralize calcium cations and calciprotein particles to prevent hydroxyapatite depositions [161]. MGP has four modified isoforms: γ-carboxylated MGP (cMGP), non-γ-carboxylated MGP (ucMGP), phosphorylated MGP (pMGP) and non-phosphorylated MGP (dpMGP) [46, 54]. The balance between cMGP and ucMGP is essential to regulate anti-microcalcification processes, which in turn regulate several factors, including the levels of vitamin K, vitamin D, retinoic acid, and extracellular calcium ions [49]. ucMGP and dpMGP are inactive, and their circulating levels are positively correlated with vascular stiffness and calcification [162]. Moreover, dp-ucMGP levels more accurately indicate vitamin K status and are associated with CAC scores. They are also considered as reliable VC biomarker outcomes in patients with CKD [163, 164]. A study has revealed that increased circulating dp-ucMGP levels independently predict VC events and all-cause mortality, with an odds ratio of 1.88, while comparing stable CVD and non-CVD subjects [165]. A similar study has demonstrated that high dp-ucMGP levels are associated with increased risks of all-cause mortality in 107 pre-dialysis patients [166]., indicating that MGP inhibition loss induces VC processes. This discrepancy is possibly due to different specific diagnostic antibodies and approaches; thus, further research is required to reach a conclusion [164, 167]. Nevertheless, as a crucial VC inhibitor, MGP and its varietal subtypes may be good diagnostic biomarkers for VC in patients with progressive CKD.

### 6.2. Fetuin-A

Various studies have revealed that low fetuin-A serum levels are inversely correlated with VC, CAC scores, and increased mortality in patients with CKD [168]. Throughout 5 years, we followed up 277 kidney transplant recipients and found that low fetuin-A levels are independently associated with aortic calcifications and a higher risk of cardiovascular events and mortality [169]. Moreover, a study on 198 patients with different stages of CKD has revealed that circulating fetuin-A significantly decreases with disease progression [170]. An additional cohort of 64 patients with moderate CKD has indicated that serum fetuin-A levels are prominently inversely correlated with CRP levels and VC-induced left ventricular hypertrophy [171]. It has been suggested that detecting specific CPP fractions from total circulating fetuin-A may serve as a better indicator of ectopic mineralization in patients with CKD than other factors, because of colloidal phase apatite binding and CPP formation. Additionally, studies have indicated that increased CPP fetuin-A levels are associated with increased aortic stiffness and high cardiovascular events in pre-dialysis patients with CKD [172, 173]. As a significant VC inhibitor, serum-circulating fetuin-A and the corresponding CPP fractions may serve as favorable diagnostic VC biomarkers.

### 6.3. Pyrophosphate

PPi is another key VC striking inhibitor, and its homeostasis is regulated by ENPP1, ANK, and TNAP. Activated ENPP1 and ANK inhibit ectopic calcification by upregulating PPi products, whereas TNAP enhances VC progression, cardiac hypertrophy, and premature death by downregulating PPi levels [66, 67]. A model of mice with CKD has shown that daily peritoneal dialysis with a solution containing PPi may significantly suppress VC [174]. Extensive soft tissue calcification was induced in ENPP1 deletion mice, particularly in tendons and ligaments tissues. Similarly, mice without ANK develop calcifications and progressive inflammatory arthritis, which are phenotypes induced by decreased PPi production [175, 176]. Clinical studies have shown that plasma PPi levels are inversely correlated with VC in patients with CKD; the degradational products of PPi increase during hemodialysis [20, 177, 178]. Furthermore, patients undergoing dialysis exhibit low levels of serum PPi; its reduction rate is related to hemodialysis duration [179]. PPi levels are determined by related metabolic enzymes, including ENPP1, TNAP, and ANK, which are essential for regulating VC processes synergistically. Therefore, in addition to PPi, the quantity and quality changes in the aforementioned related molecules may be good diagnostic parameters for VC in patients with CKD.

### 6.4. FGF23 and klotho

FGF23 is approximately a 30-KDa protein comprising 251 amino acids that synergize with its co-receptor klotho to regulate ectopic calcification. Klotho binds with FGF23 to regulate mineralization; thus, these factors are usually discussed together. It has been illustrated that FGF23 levels increase with CKD progression, and high FGF23 levels have a compensatory effect, enhancing uric phosphate excretion to respond to hyperphosphatemia [180]. Accordingly, klotho has been reported to inhibit VC processes by repressing phosphate reabsorption, resulting in phosphatidic and declined calcitriol synthesis [57, 61]. Klotho levels gradually decrease as CKD progresses and are inversely correlated with hyperphosphatemia. Serum klotho levels are reportedly significantly lower in patients with CKD than in healthy controls, while strikingly lower in CKD populations than in healthy cohorts, even in children[181]. Klotho and FGF23 levels are also related to CKD-MBD. Loss of renal klotho levels in patients with CKD-MBD is parallel to hyperphosphatemia, osteodystrophy, and vitamin D deficiency and is negatively associated with FGF23 levels. This may be attributed to the fact that decreased klotho levels limit FGF23 signaling activation, thus making patients secrete more FGF23 to compensate for the decreased klotho levels [182]. This phenomenon may explain the fact that serum FGF23 levels are strongly positively correlated whereas klotho levels are negatively associated with VC in patients with progressive CKD.

### 6.5. Sclerostin

Sclerostin is a 22-kDa glycoprotein encoded by the SOST gene, a potent soluble inhibitor of the Wnt/β-catenin and BMP signaling pathways, involved in bone metabolism by suppressing osteocyte differentiation [183]. A study using abdominal radiography on 161 patients with CKD has revealed a positive correlation between serum sclerostin levels and AAC [184]. Similarly, a study on 241 pre-dialysis patients has shown that higher sclerostin levels are significantly associated with CAC, even after adjusting for confounding factors (odds ratio, 2.18; 95% confidence interval, 1.06-4.49; and P=0.03) [185]. However, other investigations have reported lower sclerostin levels are associated with VC, and a meta-analysis has indicated sclerostin levels are not predominantly associated with all-cause and VC-related mortality risk in patients with CKD [186, 187]. This discrepancy may be attributed to population heterogeneity, different age groups, discordant CKD stages, and comorbidities. The basic pathophysiologic role of sclerostin is inhibiting mineralization metabolism. Therefore, we suspect that upregulated sclerostin may have a compensatory defensive effect to prevent further ossification; thus, it may be a clinical prognosis biomarker of VC in patients with progressive CKD. However, further basic and clinical studies are required to confirm this hypothesis.

Recent evidence has indicated that microRNAs are essential for regulating VC processes in patients with progressive CKD. Studies have suggested that miR-30B, miR-125B, miR-126, miR143, miR-145, miR-204, and miR-223 are potential biomarkers of VC in patients with CKD [188-190]. miR-30B inhibits VC processes, and its levels decrease in patients with CKD, potentially by promoting the mitochondrial membrane potential and autophagy via the mTOR signal pathway [191]. Similarly, the decreased miR-204 in patients with CKD contributes to VC via DNMT3a epigenetic regulation, forming a negative miR-204/DNMT3a regulatory circuit[192]. Complicated molecular signaling pathways are involved in VC pathogenesis, and the abovementioned circulating molecules are initiators and/or products of these pathways. These molecules are similar to the inputs and outputs of VC processes. Therefore, these pivotal regulators, engaged in VC signaling, are potential clinical biomarkers and therapeutic targets for the VC process in patients with CKD.

## 7. Therapeutic interventions for VC in patients with CKD

Visualization of hydroxyapatite depositions in arterial walls is easy owing to advanced medical imaging resolution technology, including radiography, computed tomography, and magnetic resonance imaging. However, more effective and safe interventions are required to prevent early ectopic calcification and CKD progression in cases of such depositions. Intima and media calcifications are triggered by distinctive risk factors, thus requiring different interventions according to the pathological mechanism of each calcification. Improvements in lifestyle behaviors such as strengthening exercises, weight control, and blood pressure management, inhibit intima calcification associated with atherosclerosis progression. However, we have briefly mentioned this matter in this study. We herein primarily discuss therapeutic strategies for VC in patients with CKD, including the management of calcimimetics, phosphate binder, bisphosphonates, vitamin D, vitamin K, and magnesium (Table 2).

**Table 2:** Therapeutic interventions for VC in patients with CKD.

Drugs	Targets	Comments	Ref.
25(OH)D3	CYP27B1	Activates VDR signaling and normalizes calcium levels	[246]
VS-105	VDRs	Increases VDR and klotho expression, reducing PTH expression	[218, 219]
Cinacalcet	CaSRs	EMT suppression	[199, 247]
Evocalcet	CaSRs	PTH secretion inhibition	[200]
Etelcalcetide	CaSRs	PTH expression reduction	[195]
SNF472	Calcium depositions	Inhibits the formation and growth of hydroxyapatite crystals	[229, 248, 249]
MgCO_3_	Intestinal phosphate transporters	Increases the intestinal expression of the phosphate transporters “NaPi-IIb” and “Pit-1”	[119, 250]
Sevelame	Intestinal phosphate transporters	Suppresses intestinal phosphate absorption	[251]
Zinc supplementation	NF-κB	Inhibits NF-κB activity via the GPR39-mediated upregulation of TNFAIP3	[252]
Vitamin E	Oxidative stress	Modulates adverse pro-oxidant effects	[253]
Quercetin	Oxidative stress	iNOs/p38MAPK pathway	[254]
Puerarin	Oxidative stress	Targets the NLRP3/Caspase 1/IL-1β and NF-κB pathways	[255]
sFRP5	Wnt/β-catenin	Activates the noncanonical Wnt signaling pathway	[256]
Ginsenoside Rb1	Wnt/β-catenin	Modulates PPAR-γ/Wnt/β-catenin axis	[257]
Gemigliptin	Dipeptidyl peptidase 4	Downregulates PiT-1 expression and attenuates phosphate-induced oxidative stress via phospho-AKT/PI3K signaling and Wnt signaling	[258]
KMUP-1	NO	Activates NO/cGMP/PKG pathways	[259]
Vitamin K	MGP	Upregulates MGP carboxylation and reduces serum uc-MGP levels	[224, 260-262]

### 7.1. Calcimimetics

Calcimimetics are calcium analogs and activate CaSRs, mimicking the effects of calcium on ectopic calcification inhibition. They are G-protein-coupled receptors expressed primarily in the kidneys, intestine, bones, and parathyroid glands and regulate systemic calcium homeostasis [193]. A study has suggested that CaSRs are sensitive to extracellular calcium, which acts as an agonist for the reduction of serum PTH, calcium, and phosphate levels, particularly secondary hyperparathyroidism in patients with CKD [194]. Calcimimetics interact directly with CaSRs to actuate parathyroid cells sensitive for extracellular calcium, consequently inhibiting PTH secretion, reducing bone turnover, and increasing urinary calcium excretion [195]; i.e., a “pharmacological parathyroidectomy” effect. Clinical studies have indicated that the calcimimetic cinacalcet is beneficial for CAC scores and aortic valve calcification in patients with CKD and secondary hyperparathyroidism [196-198]. In addition to inhibiting PTH secretion, cinacalcet directly prevents ectopic calcification and improves prognosis [199, 200]. Etelcalcetide, an intravenous calcimimetic, decreases both serum PTH and FGF23 levels. Another study has suggested that etelcalcetide is more effective than cinacalcet in assessing VC biochemical endpoints [195, 198, 201].

### 7.2 Phosphonates binders

Hyperphosphatemia viciously helps stimulate the transdifferentiation of VSMC into osteoblast-like cells, which is significantly associated with extensive VC and final cardiovascular events in patients with CKD [41]. Control Pi levels are beneficial to suppress VC progression. Moreover, phosphate binders are available for early clinical application [202]. Calcium-containing binders, including acetate and carbonate, and calcium-free binders, including sevelamer and lanthanum, are currently used to alleviate clinical outcomes [203]. Recent studies have suggested that patients receiving calcium-containing binders present a high risk of VC, which may be due to high calcium loading. Nevertheless, calcium carbonate has fewer side effects than other phosphate binders, though the exact mechanisms underlying calcium carbonate remains to be elucidated [204]. However, high doses of calcium should be avoided, because of the low rate of calcium-free binder-related mortality [205-207]. Furthermore, related future clinical trials may reveal various phosphate binder interventions for aortic calcification, left ventricular mass, and biochemical changes in patients with CKD [208].

### 7.3. Bisphosphonates

The bone-vascular axis theory indicates that VC processes are accompanied by osteoporosis throughout aging and CKD progression, partly because accelerated bone turnover produces calcium salts flowing into arterial walls from the bone matrix [6]. Bisphosphates (BPs) are analogs of PPi compounds, which powerfully inhibit bone turnover, and are termed “osteoclast killers.” They have been used to treat patients with VC and severe osteoporosis [209]. They are distributed to two classes: non-nitrogen-containing BPs, including clodronate and etidronate; and a new generation of nitrogen-containing BPs comprising alendronate ibandronate and pamidronate [210]. In early clinical practice, BPs were primarily utilized to treat postmenopausal osteoporosis, glucocorticoid-induced osteoporosis, and bone-related oncology complications [211]. Recent studies have discovered that alendronate effectively inhibits VC progression in patients who underwent kidney transplants [212]. Additionally, BPs are highly efficient for treating genetic disorders characterized by a reduced serum PPi/Pi ratio, including PXE, GACI, and ACDC. These disorders promote ectopic calcification in vessels and tissues [213]. Although BP treatment presents advantages, BPs have a long half-life of approximately 10 years in the human body. Therefore, further research is warranted to confirm the long-term side effects of BPs, including skeletal toxicity and osteonecrosis [214].

### 7.4. Vitamin D

Vitamin D modulates serum calcium and phosphate levels, engages in bone and extra-skeletal physiological metabolism by exerting effects on vitamin D receptors (VDRs). Activated VDRs, called calcitriol, are generated by Vitamin D 1α-hydroxylating and 25-hydroxylating. The former converts 25(OH)D to 1,25(OH)2D(calcitriol) and is primarily expressed in the kidney [31]. Studies have suggested that vitamin D deficiency is associated with VC in patients with CKD owing to 1α-hydroxylase deficiency in kidneys [215]. Nevertheless, other studies have indicated that vitamin D hypervitaminosis may unexpectedly promote aortic and coronary calcification [216]. Both high and low levels of vitamin D are associated with VC, and low-dose vitamin D administration may suppress VC progression in patients with CKD. Extensive clinical investigations have confirmed that sufficient physiological levels of vitamin D are essential for avoiding ectopic calcification. However, vitamin D homeostasis dysregulation via excess supplementation or depletion triggers VC pathogenesis [217]. In addition to vitamin D, other agonists of VDRs inhibit VC. VS-105 is a novel VDR agonist that prominently inhibits serum PTH without affecting calcium levels by directly down-regulating PTH gene expression [218]. Although VS-105 has poorly affected CKD-MBD, it induces hypercalcemia and hyperphosphatemia, potentially because it targets different VDRs [219].

### 7.5 Vitamin K

Vitamin K is a group of fat-soluble essential vitamins markedly contributing to blood clotting system modulation and a cofactor of γ-glutamyl carboxylase, which converts glutamic acid residues to Gla [220]. Studies have shown that vitamin K is also a cofactor assisting γ-carboxylation. It enhances the activity of MGP and osteocalcin, another Gla protein, via γ-carboxylation modification [221]. Vitamin K deficiency occurs among most patients with CKD and is accompanied by proportionally increased serum pump levels according to each CKD stage [222]. Clinical studies have demonstrated that daily supplementation of vitamin K is safe and increases MGP activity, slowing VC progression [223]. Similarly, numerous clinical trials have indicated that supplements of vitamin K help prevent VC in patients with CKD [163, 224, 225].

### 7.6. Magnesium

Magnesium is among the most abundant cations distributed in the human body, involving several physiologic metabolism processes. Numerous studies have revealed that low magnesium levels are associated with VC and that its supplementation may hamper VC progression [226]. Clinical trials have indicated that supplementing magnesium hydroxide for 8 weeks in patients with CKD-3/4 effectively improves calcification propensity. Several studies have recently broadly indicated that magnesium suppresses phosphate-induced calcification development and mortality in a dose-dependent manner in patients with CKD [227].

Furthermore, recent pre-clinical studies have demonstrated that other agents, including sodium thiosulfate, denosumab, SNF472, sclerostin antibody, and genetically activated klotho, also potentially inhibit VC in patients with CKD [56, 183, 228, 229]. Biotechnology progress will contribute to developing more effective and specific therapeutic agents and approaches in the future. Further studies are required to delineate pharmacological mechanisms, clinical efficacy, and safety of these agents and interventions.

## 8. Conclusions and perspectives

VC is a detrimental complication of CKD presenting a vascular aging phenotype and is induced by multifactorial damage, triggering VSMC osteogenesis. Hyper-phosphatemia, spathe, and uremia are powerful stimuli for trans-differentiating VSMCs into osteoblastic cells by upregulating transcription factors, including Runx2, Msx2, and osterix. Moreover, pro-aging factors, such as oxidative stress, SASP, LaminA mutation, and klotho significantly accelerate VC processes. Dysregulated metabolites in patients with progressive CKD are proven to contribute to VC; however, further research is warranted to investigate various questions and mechanisms. For instance, which metabolites are primarily responsible for triggering the transformation of VSMCs? Are there any other types of cells, except VSMCs, such as mesenchymal stem cells, fibroblasts, and inflammatory cells, affected by these metabolites, and how do they affect VC processes? What are the pivotal signal pathways that respond to these disordered metabolites, and how do relate intercrossing signals affect osteogenesis in vessel walls? More confounding questions arise when aging exacerbates CKD and aggravates VC processes. How do these pro-aging factors affect CKD and VC processes? It is essential to illustrate the way the uremic milieu in CKD affects aging and VSMC osteogenesis. Studies have reported that klotho loss induces renal tubule dysfunction and consequently accelerates VC processes. However, the relationship between the klotho-FGF23 signaling pathway, CKD metabolism, and VC interaction should be further elucidated. FGF23 and klotho are similar to endocrine hormones that regulate bone mineralization; thus, the unequivocal mechanisms involving the bone-vascular axis mediated by FGF23-klotho should be fully elucidated. Animal models are available to mimic VC pathophysiologic processes *in vivo*. Therefore, aging combined with kidney tissue-specific gene modification mice models should be established to explore VC mechanisms in patients with CKD.

VC is a heterogeneous and multifactorial process. Therefore, identifying diagnostic biomarkers for VC combined with CKD is arduous. Detecting a single biomarker may not accurately reflect VC processes in patients with CKD, because sensitivity and specificity are marked parameters for biomarker identification. Therefore, biomarker panels should be developed. In patients with CKD progression, disordered metabolites including uremia and hyperphosphatemia, significantly aggravate cell senescence. Consequently, senescent VSMCs release large quantities of apoptosis-resistant cytokines, serving as vicious accelerators of VC processes. Recent studies have revealed that senolytic intervention is a valid method to clean senescence cells [230]. Thus, senolytic reagents may be a new strategy for suppressing VC progression by inducing early senescence VSMCs senolysis. Epigenetic modifications have been reported to be involved in pathophysiologic processes and therapeutic applications [231]. Studies have shown that aging and VC processes are regulated by epigenetic modification, including microRNAs and histone post-translational modulators [232, 233]. However, novel questions should be addressed in future studies. For example, what are the key aging epigenetic modulators activated in patients with CKD? Are there any existing integrative signal pathways linking epigenetic modulation with aging and osteogenic signaling in patients with progressive CKD? Finally, how do the aforementioned signaling molecules crosstalk with each other? Further research will allow for a better understanding of VC pathogenesis and help us renew therapeutic applications.
